# Low Bone Mass in Ambulatory Spinal Muscular Atrophy: A Proactive Approach for an Often-Overlooked Impairment

**DOI:** 10.3390/jcm13051336

**Published:** 2024-02-27

**Authors:** Caitlin Trancho, Bailey Stickney, Stacy Kinirons, David Uher, Cara H. Kanner, Ashwini K. Rao, Michael P. McDermott, Carol Ewing Garber, Darryl C. De Vivo, Jacqueline Montes

**Affiliations:** 1 Department of Rehabilitation and Regenerative Medicine, Columbia University Irving Medical Center, New York, NY 10032, USAjm598@cumc.columbia.edu (J.M.); 2 Departments of Biostatistics and Computational Biology and Neurology, University of Rochester, Rochester, NY 14627, USA; michael_mcdermott@urmc.rochester.edu; 3 Department of Biobehavioral Sciences, Teachers College, Columbia University, New York, NY 10027, USA; 4 Department of Neurology, Columbia University Irving Medical Center, New York, NY 10032, USA

**Keywords:** atrophy, spinal muscular, bone density, osteoporosis, adult, young adult, mobility limitation, physical activity

## Abstract

**Background:** Individuals with spinal muscular atrophy (SMA) are at risk for low bone mass (LBM). The objectives of this study were to compare bone mineral density (BMD) in ambulatory SMA and control participants, identify LBM, and evaluate the associations of function and physical activity (PA) with LBM. **Methods:** Thirty-five children and adults, nineteen SMA and sixteen healthy controls, participated. Dual-energy absorptiometry determined BMD, T-scores, and Z-scores. The six-minute walk test (6MWT) and Timed Up and Go (TUG) assessed function. The International Physical Activity Questionnaire Short Form (IPAQ-SF) evaluated PA. **Results:** Group comparisons and factors associated with BMD were analyzed. Area under the receiver operating characteristic (ROC) curve (AUC) assessed the ability to identify individuals with LBM. SMA participants had lower BMD (*p* < 0.001) and increased odds of having LBM relative to controls (OR = 16.7; 95%CI: 1.8–152.8; *p* = 0.004). **Conclusions:** Ten SMA and one control had LBM. Z-score was associated with 6MWT (r_s_ = 0.65; *p* < 0.001) and TUG (r_s_ = −0.61; *p* < 0.001). IPAQ-SF and Z-score were weakly associated (r_s_ = 0.36, *p* < 0.03). 6MWT (AUC: 0.80; 95% CI: 0.65–0.94; *p* = 0.006) and TUG (AUC: 0.85; 95% CI: 0.71–0.98; *p* = 0.002) identified individuals with LBM. Function, assessed by the 6MWT and TUG, is associated with BMD and shows promise for use in identifying individuals with LBM.

## 1. Introduction

Spinal muscular atrophy is a hereditary genetic disorder caused by a deletion mutation on the survival motor neuron 1 (*SMN1*) gene [1]. The *SMN2* gene, an imperfect backup, is unable to offset the *SMN1* deletion mutation, resulting in decreased production of SMN protein in all body tissues [2]. A deficit of SMN protein leads to motor neuron cell loss in the spinal cord, causing progressive muscle weakness and atrophy [1].

SMA classification has historically been determined by disease severity and maximum milestones achieved [3]. Individuals with more *SMN2* gene copies can produce greater amounts of functional SMN protein and exhibit a milder phenotype. SMA Type II is milder than SMA type I, presenting after 6 months of age. Individuals with SMA Type II sit independently. SMA Type III, the mildest phenotype, presents after 18 months of age. These individuals walk independently; however, this level of functional mobility can be lost over time. Individuals with SMA type III have the greatest functional capacity, subsequently increasing their risk for falls and injury [4].

The goal of the three currently FDA-approved disease-modifying therapies is to increase SMN protein to improve function. Results from studies leading to the approval of these therapies demonstrated increases in functional SMN protein and decreased symptom severity [5,6,7,8]. When treatment is initiated early, ideally in the pre-symptomatic stage, motor development may be more typical, even in individuals with fewer *SMN2* copies [9]. Treatment with disease-modifying therapies leads to improved survival, an improved ability to achieve motor milestones, and increased functional mobility and independence [10]. Nusinersen, administered intrathecally, targets motor neurons to increase SMN availability and improve motor function [8,9,11,12]. While these therapies are not a cure, they changed the prognosis for SMA from a fatal diagnosis to one of chronicity.

In typically developing populations, sufficient mechanical loading must occur throughout development and into adulthood to develop and maintain adequate bone mass [13,14,15]. In children, physical exercise stimulates bone production, while in adults, it counteracts the resorption of bone that naturally occurs with age [13,15]. Increased physical activity throughout the lifespan has been shown to have positive effects on bone mass in healthy individuals [16,17,18]. In SMA types II and III, increased motor function is associated with increased total hip bone mineral density (BMD) Z-scores [19]. Decreased function and physical activity place individuals with SMA at risk for decreased BMD, increasing their risk for fractures and associated negative sequelae.

Children with SMA have lower BMD scores than children with other pediatric neuromuscular disorders despite having similar limitations in strength and function [20]. Non-ambulant children with SMA have lower BMD [20] and significantly lower subtotal (whole body minus the head) BMD and lumbar spine BMD Z-scores than children with SMA type III [21]. In a pediatric-focused study, low BMD was not observed in younger participants with SMA types II and III (*n* = 12) [22]. Low BMD was defined as total body BMD more than two standard deviations (SDs) below the mean from a data set of healthy Dutch children and adolescents. The two young adults in the study, both with SMA Type III, lost independent ambulation approximately 3.5 years prior and were the only participants with low BMD. The authors concluded that the decreased BMD in these two participants was likely related to age, as they were the oldest individuals in the cohort [22]. Little research has been published regarding BMD in ambulatory individuals with SMA.

The purpose of this study was to (1) compare BMD in ambulatory children and adults with SMA to that in healthy control participants, (2) identify individuals with LBM, and (3) examine the associations of function and physical activity with bone density.

## 2. Materials and Methods

Ambulatory children and adults with SMA and healthy controls were invited to participate in this observational study. This is a secondary analysis of data from a previous study, registered with ClinicalTrials.gov (NCT02895789), that examined muscle oxygen uptake during exercise [23]. A total of 35 participants were included—19 ambulatory individuals with SMA and 16 age-matched control participants. Participants were required to be at least 12 years old, able to walk 25 m without assistance, and ride a stationary cycle ergometer. Participants were excluded if they used an investigational medication intended for treatment of SMA. Participants or guardians provided informed consent, approved by the Columbia University Irving Medical Center Institutional Review Board.

### 2.1. Six-Minute Walk Test (6MWT)

The six-minute walk test (6MWT) is an objective measure of functional exercise capacity. It evaluates the maximum distance an individual walks in 6 min on a 25 m linear course. It is a valid measure of functional exercise capacity and ambulatory function in SMA [24]. The total distance walked over the 6 min time period was recorded.

### 2.2. The Timed Up and Go (TUG)

The Timed Up and Go (TUG) test is an objective measure of mobility and an assessment tool for fall risk. Individuals are timed as they stand up from a standard armchair, walk 3 m, turn around, then walk back to the chair and sit. The timer stops when the individual is seated. The TUG test was validated in children and adults and is a reliable measure of function in SMA [25].

### 2.3. The International Physical Activity Questionnaire—Short Form (IPAQ-SF)

The IPAQ-SF was used to assess physical activity over the previous seven days. The IPAQ-SF is composed of seven questions from four domains of physical activity, including vigorous and moderate physical activity, walking physical activity, and sitting time. A weighted total from vigorous physical activity, moderate physical activity, and walking physical activity is calculated to produce a total amount of physical activity in MET-min/week (MET-min/week = MET level × minutes of activity/day × days per week). MET levels are weighted based on intensity of activity. IPAQ-SF was validated to evaluate physical activity in individuals 15–69 years old [26,27] and in several neuromuscular populations, including Charcot–Marie–Tooth disease and limb–girdle muscular dystrophy [28].

### 2.4. Dual X-ray Absorptiometry (DEXA)

DEXA measures bone density using low-power X-ray beams. It is a safe and reliable measure that was validated as a diagnostic tool for osteoporosis. DEXA scans produce T- and Z-scores, which are used to identify LBM dependent on age [29]. A T-score calculated from DEXA measurements compares the participant’s total BMD to a healthy 30-year-old control. A Z-score calculated from DEXA measurements compares the participant’s total BMD to age- and sex-matched controls. In individuals 50 years old or older, a T-score < −1 indicates LBM. In individuals younger than 50 years old, a Z-score < than −2 indicates LBM. Additionally, subtotal BMD (whole body minus head) and several regional BMD scores were calculated.

### 2.5. Statistical Methods

Mann–Whitney tests were used for group comparisons given the non-normal data distribution and small sample size. Spearman rank correlation coefficients were used to determine which factors were associated with BMD. T-scores below −1.0 for those > 50 years old and Z-scores below −2 for those < 50 years old were used to identify individuals with LBM. A univariate logistic regression analysis was used to determine an Odds Ratio (OR). The area under the non-parametrically estimated receiver operating characteristic (ROC) curve (AUC) and its associated 95% confidence interval was used to assess the ability of the IPAQ-SF, 6MWT, and TUG to identify individuals with LBM. Thresholds for LBM in all participants, those with SMA and controls, were determined based on maximizing the Youden index or, equivalently, the sum of sensitivity and specificity.

## 3. Results

The clinical characteristics of the participants are presented in Table 1. The average age for the SMA and control groups was 32.9 years old (Range 12.7–56.8, SD = 15.4) and 28.1 years old (Range 13.3–53.5, SD = 14.4), respectively, and the participants in each group were predominantly male. There were no significant differences in age or sex between the groups. In the 13 participants receiving nusinersen, the average duration of treatment was 1 year (SD = 0.3). In the SMA group, 2 participants were taking a vitamin D supplement, and 1 participant took medication for osteoporosis, while no controls reported taking either.

On average, participants with SMA walked approximately half the distance of control participants during the 6MWT (*p* < 0.001) (Table 1). Participants with SMA completed the TUG in an average of 21.3 s, while control participants completed it in an average of 4.3 s (*p* < 0.001). Participants with SMA reported a median Total METmin/week nearly half that of controls on the IPAQ-SF (*p* = 0.05). Median sitting time in participants with SMA was 480 min (IQR = 240–600), while median sitting time in controls was 420 min (IQR = 360–480).

Participants with SMA had significantly lower BMD than control participants in most regions measured (Table 2). The spine was the only region of the body that was not significantly different between groups. Subtotal BMD, extremity BMD, and T and Z-scores were significantly lower in participants with SMA compared to control participants.

**Figure 1:**
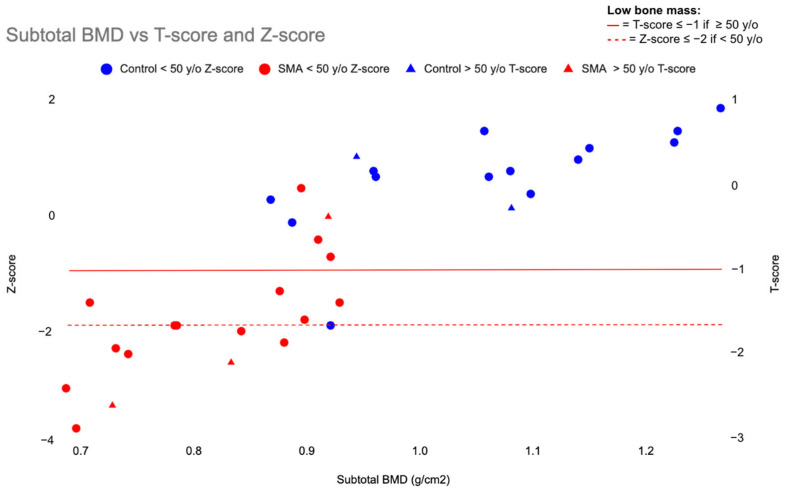
Subtotal BMD vs. T-score and Z-score. Correlation between subtotal BMD and both Z-score or T-score in participants with SMA and control participants. Participants with SMA are plotted in red. Control participants are plotted in blue. Participants under 50 years old (circles) are plotted versus Z-score; those falling on or below the dashed red line have LBM. Participants 50 years of age or older (triangles) are plotted versus T-score; those falling on or below the solid red line have LBM. BMD = bone mineral density, SMA = spinal muscular atrophy, LBM = low bone mass, y/o = years old, g/cm^2^ = grams per centimeter squared.

Standard scores were calculated for subtotal BMD in SMA and controls. T-scores were used for those > 50 years old and Z-scores for those < 50 years old. Ten (52.6%) participants with SMA, most (n = 8) of whom were less than 50 years old, and 1 (6.3%) control participant met the criteria for LBM, revealing participants with SMA had 16-times greater odds (OR = 16.7, 95% CI 1.8–152.8) of having LBM (Figure 1).

**Figure 2:**
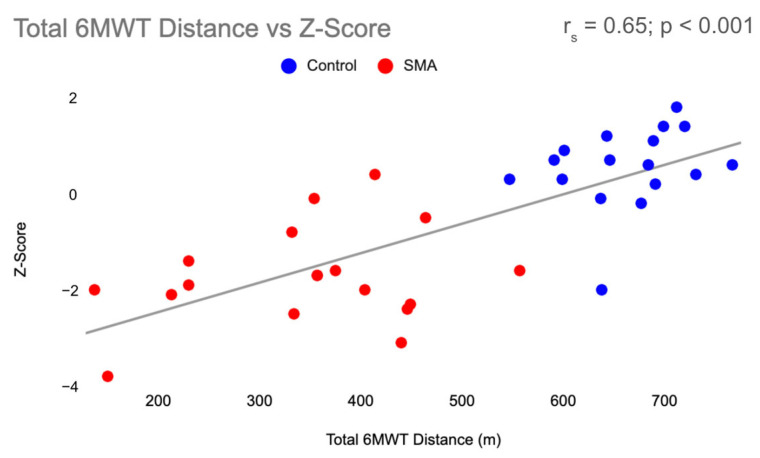
Total 6MWT Distance vs. Z-score. Correlation between total distance walked in meters during the 6MWT and Z-score, with SMA participants in red and control participants in blue. 6MWT = six-minute walk test, SMA = spinal muscular atrophy, m = meters.

**Figure 3:**
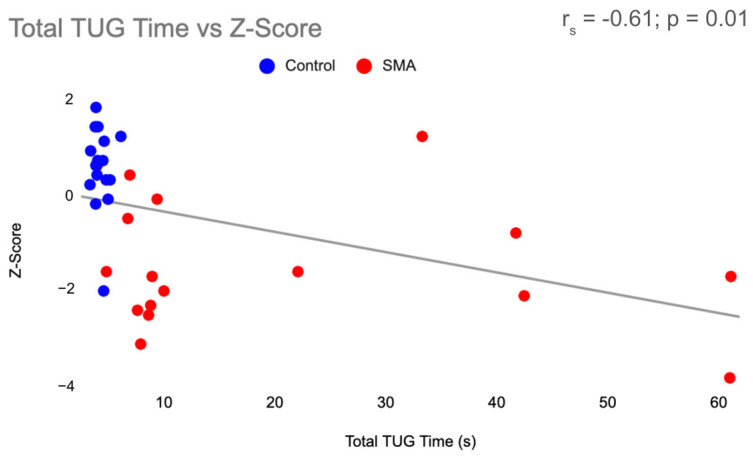
Total TUG Time vs. Z-score. Correlation between time to complete the TUG and Z-score, with SMA participants in red and control participants in blue. TUG = Timed Up and Go, SMA = spinal muscular atrophy, s = seconds.

**Figure 4:**
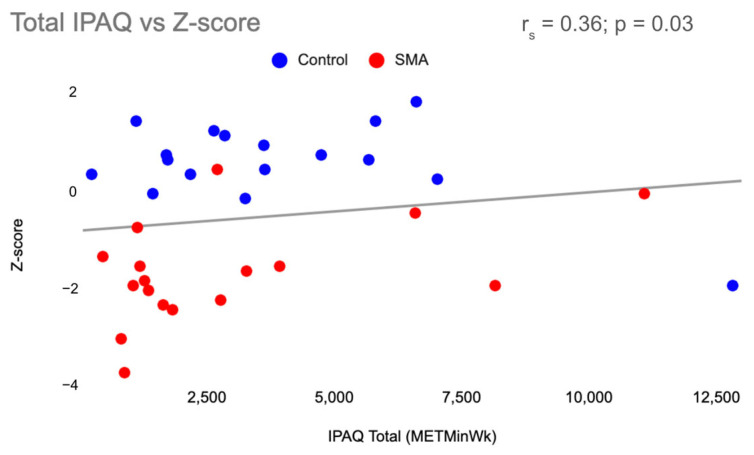
Total IPAQ-SF vs. Z-score. Correlation between total METMin/Wk reported on the IPAQ-SF and Z-score, with SMA participants in red and control participants in blue. IPAQ = International Physical Activity Questionnaire, SMA = spinal muscular atrophy, METMin/Wk = metabolic equivalent of task minutes per week.

Function, measured by the 6MWT and TUG, was found to be associated with BMD in all participants. There was a moderately positive correlation (r_s_ = 0.65; *p* < 0.001) between 6MWT and Z-Score (Figure 2) and a moderately negative correlation (r_s_= −0.61; *p* = 0.01) between TUG and Z-score (Figure 3). Physical activity and Z-score were weakly associated (r_s_ = 0.36; *p* = 0.03) (Figure 4).

**Figure 5:**
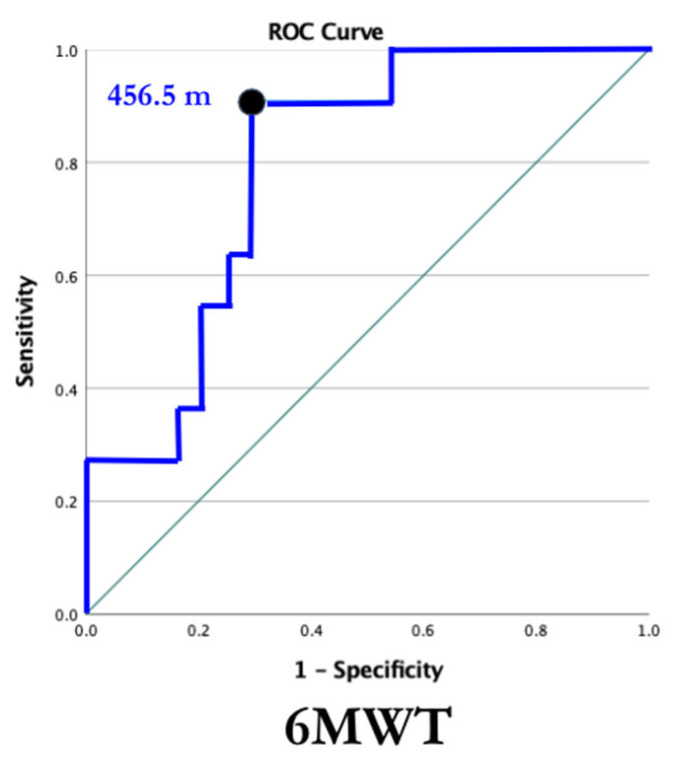
6MWT ROC curve. Receiver operating characteristic curve for the 6MWT. The threshold for LBM in all participants was determined based on maximizing the Youden index, which is identified by the dot. AUC demonstrates the ability of the 6MWT to discriminate between individuals with and without LBM. ROC = receiver operating characteristic, 6MWT = six-min walk test, AUC = area under curve, LBM = low bone mass. AUC = 0.80, 95% CI [0.65–0.94], *p* = 0.006.

**Figure 6:**
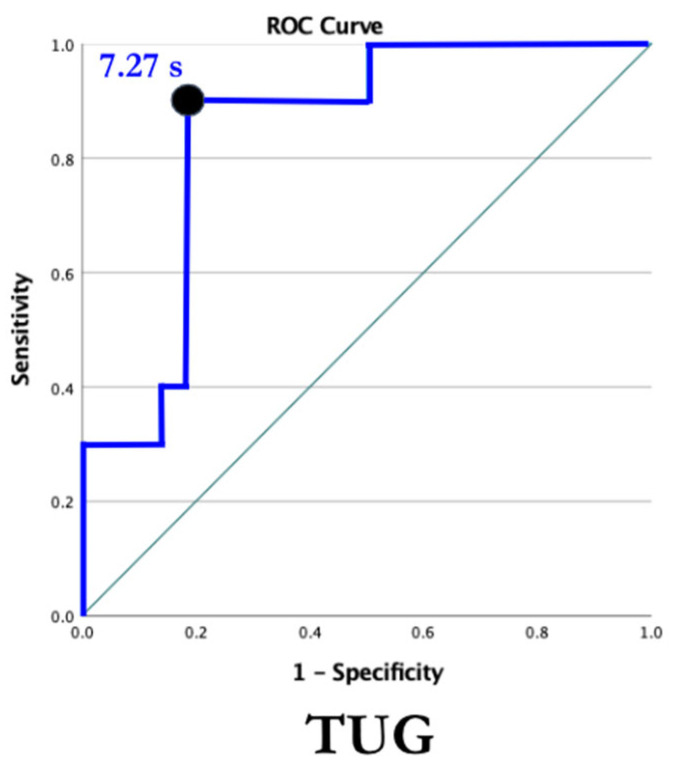
TUG ROC curve. Receiver operating characteristic curve for the TUG. The threshold for LBM in all participants was determined based on maximizing the Youden index, which is identified by the dot. AUC demonstrates the ability of the TUG to discriminate between individuals with and without LBM. ROC = receiver operating characteristic, TUG = Timed Up and Go, AUC = area under curve, LBM = low bone mass. AUC = 0.85, 95% CI [0.71–0.98], *p* = 0.002.

Receiver operating characteristic curves were used to examine the ability of the 6MWT and TUG to identify individuals with LBM in all participants (Figure 5 and Figure 6). With an AUC of 0.80, the 6MWT can be categorized as good at discriminating between individuals with and without LBM (Figure 5). The ROC curve for the TUG revealed an AUC of 0.85, indicating the TUG test was also good at discriminating between individuals with and without LBM (Figure 6). Cut-off values were identified for both measures of function using data from the ROC curves. The chosen threshold from the ROC curve (Figure 2) for the 6MWT associated with LBM (Z-score < −2) in SMA and healthy controls was 456.5 m (sensitivity, 91%; specificity, 71%). The chosen threshold for TUG (Figure 3) associated with LBM (Z-score < −2) in SMA and healthy controls was 7.27s (sensitivity, 90%; specificity, 82%).

## 4. Discussion

Prior to this study, the published literature focused on bone density in pediatric, untreated, non-ambulatory SMA populations. This cross-sectional analysis of a primarily adult cohort with SMA complements prior findings of low bone density in pediatric SMA [20,21,22]. In our study, ambulatory children and adults with SMA have decreased BMD compared to age- and sex-matched controls and 16-fold greater odds of having LBM. Additionally, function, as measured by the 6MWT and TUG, is associated with BMD. Scores reported from these validated measures of function can discriminate between individuals at risk for LBM. These validated measures of exercise capacity, mobility, fall risk, and physical activity show promise as screening tools for LBM in SMA. Further work is needed in an SMA-specific cohort to determine the precise thresholds for LBM in this population.

### 4.1. Six-Minute Walk Test

In SMA, the 6MWT was validated to capture functional changes associated with motor weakness, endurance, and fatigue demonstrated by positive associations with Hammersmith Functional Motor Scale Expanded (HFMSE) scores, lower extremity MMT, and exercise capacity (e.g., peak oxygen uptake, VO2peak) [24]. Individuals with SMA were shown to walk significantly shorter distances in the sixth minute of testing compared to the first, demonstrating a unique progressive fatigue pattern [30]. The referenced norm distance walked for a healthy male between ages 20 and 59 is 736 m [31]. Meters walked on the 6MWT may provide insight into an individual’s ability to participate at the community level. Individuals with significantly decreased scores on the 6MWT should be considered for further screening for LBM. With further research in an SMA cohort, a cutoff point may be determined more precisely to discriminate between those with and without LBM.

### 4.2. Timed Up and Go Test

The TUG test is a tool that captures functional changes associated with strength, balance, and overall mobility [25]. In healthy older adults, a time > 13.5 s indicates fall risk [32], and falls increase the risk for subsequent sequelae (i.e., fracture and injury). The TUG is associated with HFMSE, total leg strength, 10 m walk/run, and the 6MWT and is a useful outcome measure in SMA [25]. Individuals who require significant time to complete the TUG should be considered for further screening for LBM. Additional research is needed to ascertain more precisely the time to complete the TUG that can identify those with LBM in an SMA-specific cohort. It is important for clinicians to recognize the high risk associated with increased time to complete the TUG, implicating both risk for falls and potentially impaired bone density.

The 6MWT and TUG were identified as reliable and valid measures of function in SMA and are easily administered [24,25]. A variety of clinicians can administer either measure, increasing the likelihood that those at risk for LBM are identified. With false positive rates of 29% and 18% for 6MWT and TUG tests, respectively, there are low-risk implications associated with falsely identifying an individual at risk for LBM, given the follow-up would simply be further screening. There is high value in identifying LBM early on to reduce the risk for subsequent sequelae from a young age. Both measures of function should be seen as valuable tools to monitor LBM in the future.

### 4.3. Bone Density across the Lifespan

In typically developing populations, BMD increases through adolescence, often peaking between 30 and 39 years old, then gradually declines with age [33]. From both the results of this study and the previous literature, it is evident that BMD in SMA is altered from what we expect to see across various ages [20,34,35,36]. This cross-sectional study is only a window into bone density in ambulatory SMA. Future studies are needed to examine the trajectory of bone health and development in SMA. A longitudinal study evaluating BMD 18 months apart in children with type II and type III SMA found that bone resorption markers were above normal at each visit [34]. Importantly, BMD decreased at the 18-month follow-up despite Vitamin D3 therapy and increased dietary calcium intake [34]. Another pediatric study looking at bone biomarkers found normal bone resorption with decreased bone formation in non-treated SMA type II and type III, resulting in reduced bone mass over two years [19]. While these studies demonstrate conflicting findings at a metabolic level, they reinforce the findings of altered bone development in SMA.

### 4.4. Extrinsic Mechanisms

It is known that repetitive mechanical loading of bone promotes osteogenesis [37]. To prevent and/or reduce the deleterious effects of osteoporosis, the National Osteoporosis Foundation recommends regular weight-bearing and muscle-strengthening exercises [38]. Individuals with SMA type III have been ambulatory at some point in their lifetime and, therefore, have increased opportunities to take part in weight-bearing activity compared to non-ambulatory individuals with SMA. This study found that ambulatory individuals with SMA decreased bone density compared to their age- and sex-matched peers. There are a variety of possible explanations, including decreased physical activity that may reduce the overall osteogenic promoting opportunities.

### 4.5. Intrinsic Mechanisms

There is some evidence to support the presence of a role of SMN in bone development and resorption in vitro, preclinical, and clinical studies. From bone marrow derived from humans and mice, Kurihara et al. identified a relationship between osteoclast stimulating factor (OSF) and SMN protein, suggesting further research into the effect of SMN protein on bone development [39]. SMA mouse models were also used to examine this relationship. In pre-symptomatic 4-day-old mice, there was no measured change in trabecular and cortical bone thickness or difference in BMD from control mice [40]. However, decreased chondrocyte density was reported and associated with decreased longitudinal bone growth [40].

In symptomatic 4-month-old mice, trabecular and cortical bone were thin at the proximal growth plates. Increased osteoclast formation and bone resorption were measured, along with decreased osteoblast differentiation marker levels, suggesting a role of SMN protein in bone remodeling [41]. Together, these mouse models support Kurihara et al.’s findings that SMN protein, or lack thereof, may play a role in the signaling mechanisms of bone growth and remodeling [39].

Blood biomarkers were used clinically to examine bone metabolism in SMA and reveal conflicting alterations [19,42]. While blood calcium in SMA children was normal, Vai et al. found elevated bone resorption marker levels (CTx) and a significant negative association between PTH and 25-OH D levels [42]. Decreased 25-OH D levels stimulate increased parathyroid hormone secretion, resulting in a net negative effect on bone mass. Kroksmark et al. found decreased bone formation marker levels (BALP) and normal bone resorption marker levels (CTx) [19]. Although these are differing mechanisms, both studies reveal changes in bone metabolism that result in loss of bone mass in SMA. One must question the role of SMN and the impact of the lack of SMN on bone formation and resorption.

### 4.6. Future of BMD in SMA

Both intrinsic and extrinsic factors play a role in BMD in SMA and should continue to be studied because it is documented that both resistance training and weight-bearing activity influence BMD in healthy populations [43]; future research is needed to investigate specific types of activity, and their effect on BMD in SMA. Given the significant correlation found between muscle mass and bone tissue health in children with SMA [35], there is promise in exercise-based interventions to improve bone health in SMA. From a rehabilitation standpoint, this is inclusive of physical interventions and forms of therapeutic exercise. Progress could be monitored at regular intervals by biomarker trends, periodic DEXA scans, and functional outcome measures.

Decreased BMD increases fracture risk in all populations [44]. A greater risk of fracture associated with altered bone mass was mostly observed in non-ambulatory children with SMA [34,36,42,45]. Our study supports the addition of ambulatory children and adults to the at-risk population in SMA. Currently, The National Osteoporosis Foundation and SMA Standard of Care Guidelines recommend Vitamin D3 therapy and calcium monitoring for individuals at risk for LBM [1,38]. However, in SMA, Vitamin D3 therapy and increased dietary calcium intake might not be adequate [34,42]. Because of the negative implications associated with low BMD, future standard of care guidelines should emphasize the risk for LBM in this population and identify management strategies to mitigate negative sequelae and create a more comprehensive standard of care.

### 4.7. Limitations

The data analyzed for this study were initially collected for a study looking at muscle oxygen uptake and fatigue in SMA. Therefore, when obtaining a subjective history from the participants, a history of falls and fractures was not collected. An instrumented measure of physical activity, along with the IPAQ, may provide more information about the association between BMD and physical activity. Due to our small sample size, analyses, including correlation analyses, were completed in the whole cohort and included both control and SMA participant data. As ROC curves were constructed using data from the entire cohort, the calculated cut-off scores and screening performance for the 6MWT and TUG might not strictly apply to the population of ambulatory individuals with SMA.

## 5. Conclusions

Ambulatory children and adults with SMA have decreased BMD compared to age- and gender-matched controls and 16-fold greater odds of having LBM. Function, measured by the 6MWT and TUG, is associated with BMD in this population. Decreased function and physical activity place individuals with SMA at risk for LBM. Decreased BMD increases fracture risk in all populations. In a population that is already at risk for falls/fractures, identification and proactive clinical management are imperative. In addition to the 2018 SMA Standard of Care guidelines that include vitamin D monitoring and supplementation, the 6MWT and TUG show promise as screening tools for LBM. A variety of clinicians can administer these quick and inexpensive outcome measures and should do so on a regular basis. Blood biomarkers and DEXA scans can also be incorporated to monitor bone mass over time [19,42]. Once identified to be at elevated risk, individuals should undergo further screening and management. Clinical management in the rehabilitation setting should include functional training, weight-bearing and resistive exercise, and fall prevention strategies to mitigate risk for subsequent sequela.

## Figures and Tables

**Table 1:** Participant characteristics.

Participant Characteristics	SMA (*n* = 19) Mean + SD	Control (*n* = 16) Mean + SD
Age (years)	32.9 ± 15.4	28.1 ± 14.4
Sex (% male)	73.7%	81.3%
Nusinersen Duration (years)	1.0 ± 0.3	-
Vitamin D Supplement (*n*)	2	0
Medication for Osteoporosis (*n*)	1	0
6MWT Distance (meters)	342.1 ± 113.5	670.3 ± 50.8
TUG Time (seconds)	21.3 ± 19.9	4.3 ± 0.7
Physical Activity: IPAQ-SF Total ^1^ (METmin/week)	1644.0 (2875.0)	3437.3 (3934.4)
Physical Activity: IPAQ-SF Sitting Time ^1^ (METmin/week)	480.0 (360.0)	420.0 (120.0)

Statistics presented as mean (SD), median (IQR), or percentage or median. SMA = spinal muscular atrophy, *n* = number, SD = standard deviation, 6MWT = six-minute walk test, TUG = Timed Up and Go, IPAQ = International Physical Activity Questionnaire, METmin/week = metabolic equivalent of task minutes per week, IQR = Interquartile Range. ^1^ Physical activity and sitting time presented as median (IQR).

**Table 2:** Mean DXA results.

DXA Variable	SMA Mean + SD	Control Mean + SD	*p*-Value
Subtotal BMD (g/cm^2^)	0.8194 ± 0.0900	1.0579 ± 0.1249	<0.001
T-score	−2.159 ± 1.2289	−0.025 ± 1.3128	<0.001
Z-score	−1.729 ± 1.0475	0.563 ± 0.8793	<0.001
Left Arm BMD (g/cm^2^)	0.7016 ± 0.0890	0.8303 ± 0.1125	<0.001
Right Arm BMD (g/cm^2^)	0.7070 ± 0.0859	0.8260 ± 0.0926	<0.001
Thoracic Spine BMD (g/cm^2^)	0.8269 ± 0.1287	0.8976 ± 0.1542	0.18
Lumbar Spine BMD (g/cm^2^)	1.0255 ± 0.1392	1.0916 ± 0.1780	0.37
Left Leg BMD (g/cm^2^)	0.9292 ± 0.1068	1.2818 ± 0.1689	<0.001
Right Leg BMD (g/cm^2^)	0.9214 ± 0.0979	1.280 ± 0.1753	<0.001

Values presented as mean BMD in g/cm^2^ + SD, or T or Z-score as appropriate. *p*-values were obtained from Mann–Whitney tests. DXA = dual X-ray absorptiometry, SD = standard deviation, BMD = bone mineral density, g/cm^2^ = grams per centimeter squared.

## Data Availability

The data presented in this study are available upon request from the corresponding author. The data are not publicly available due to privacy reasons.
